# Supplementary material for: Research on short-term power load forecasting based on VMD and GRU
Source: PLoS One. 2024 Jul 11;19(7):e0306566. doi: 10.1371/journal.pone.0306566 (PMC11239006; doi:10.1371/journal.pone.0306566)
Supplement: Attachment — Submitted filename: response.docx [file pone.0306566.s001.docx]

Reviewer #1:

1. Answer： The paper is of poor quality and cannot be published in its current form, and the proposed method is not innovative enough.

Response: Thank the reviewers for their valuable suggestions. We have improved the content of the paper, but our previous writing was not comprehensive enough, and we have added some experiments to prove the innovation of our experiments.

2. Answer： The quality of the picture is poor, please modify it.

Response: Thank the reviewers for their valuable suggestions. We redrew the model drawing to improve the picture quality, and the specific modifications are shown in Figure 1.

Figure 1 VCAG model

3 Answer： Grammar issues are an important issue currently.

Response: Thank the reviewers for their valuable suggestions. We have corrected the grammar errors and also asked my supervisor to help review them.

1. De Giorgi, M.G.; Congedo, P.M.; Malvoni, M. Photovoltaic power forecasting using statistical methods: Impact of weather data.IET Sci. Meas. Technol. 2014, 8, 90–97.
2. Sansa, I.; Boussaada, Z.; Bellaaj, N.M. Solar Radiation Prediction Using a Novel Hybrid Model of ARMA and NARX. Energies 2021, 14, 6920
3. Jiang, Y.; Zheng, L.; Ding, X. Ultra-short-term prediction of photovoltaic output based on an LSTM-ARMA combined model driven by EEMD. J. Renew. Sustain. Energy 2021, 13, 046103.

4. Answer： There are many formatting errors in references.

Response: Thank the reviewers for their valuable suggestions. We have made modifications to the references and added some additional ones.

5. Answer： The comparison method is not novel enough and it is difficult to demonstrate the effectiveness of the proposed method.

Response: Thank the reviewers for their valuable suggestions. we have modified the comparison method and added several methods for comparison.

|  | MAE | RMSE | R2 |
| --- | --- | --- | --- |
| LSTM | 66.90731364489719 | 86.26923750627041 | 0.5411458782684222 |
| GRU | 56.66997869155141 | 76.1676953359481 | 0.6563376596261866 |
| BERT^[22]^ | 52.31486598143588 | 72.1107546789745 | 0.6966775446689624 |
| BP[23] | 53.50414850471025 | 74.9748469241702 | 0.6534276640279069 |
| CNN-LSTM[24] | 52.22276598143925 | 71.0170796348745 | 0.6890515927027088 |
| CNN-GRU | 51.65861588786915 | 74.65942015095665 | 0.6423120182754858 |
| VMD-CNN-GRU | 11.602667400729006 | 14.06421909802147 | 0.9878046585346968 |
| VMD-CNN-AM-GRU | 6.0957062480374 | 8.340781585166098 | 0.9957108008069071 |

6. Answer： Insufficient literature analysis and few references.

Response: Thank the reviewers for their valuable suggestions. We have added additional references and compared our algorithm with the algorithms in the literature.

Reviewer #2: This article requires major revisions.
1. Answer：the author needs to revise the manuscript English and made clearly the contributions, as more research is already carried out in this area.

Response: Thank the reviewers for their valuable suggestions. We have revised some unclear areas in the initial draft and highlighted the innovation of this article.
2. Answer The references are not in a correct format even including their citation.

Response: Thank the reviewers for their valuable suggestions. There were indeed errors in the previous literature. We have modified the reference format and added some new references.
3. Answer: perform statistical analysis, log loss.

Response: Thank the reviewers for their valuable suggestions. We have added the calculation of losses in the article and provided the losses during the training process.

4. Answer: Compare it with BERT Model.

Response: Thank the reviewers for their valuable suggestions. We used the BERT model for prediction and included the scores in the evaluation table.

|  | MAE | RMSE | R2 |
| --- | --- | --- | --- |
| LSTM | 66.90731364489719 | 86.26923750627041 | 0.5411458782684222 |
| GRU | 56.66997869155141 | 76.1676953359481 | 0.6563376596261866 |
| BERT^[22]^ | 52.31486598143588 | 72.1107546789745 | 0.6966775446689624 |
| BP[23] | 53.50414850471025 | 74.9748469241702 | 0.6534276640279069 |
| CNN-LSTM[24] | 52.22276598143925 | 71.0170796348745 | 0.6890515927027088 |
| CNN-GRU | 51.65861588786915 | 74.65942015095665 | 0.6423120182754858 |
| VMD-CNN-GRU | 11.602667400729006 | 14.06421909802147 | 0.9878046585346968 |
| VMD-CNN-AM-GRU | 6.0957062480374 | 8.340781585166098 | 0.9957108008069071 |

5. Answer: Revise the major sections accordingly.

Response: Thank the reviewers for their valuable suggestions. We have adjusted the content of some chapters and also modified the content of some chapters.
6. Answer: extensive English changes requires.

Response: Thank the reviewers for their valuable suggestions.We have corrected the grammar of English and polished it again
7. Answer: correct the shape of the article. it seems a report rather than a research article.

Response: Thank the reviewers for their valuable suggestions. We have adjusted the chapters and also made some modifications to make the article more like an academic paper
