# Supplementary material for: Research on short-term power load forecasting based on VMD and GRU
Source: PLoS One. 2024 Jul 11;19(7):e0306566. doi: 10.1371/journal.pone.0306566 (PMC11239006; doi:10.1371/journal.pone.0306566)
Supplement: Attachment — Submitted filename: response.docx [file pone.0306566.s002.docx]

1.Answer：Additionally, one area of improvement is the use of "we" in some sentences, which may slightly detract from the formality of the writing. Shifting to an "agentless passive" construction can enhance formality. For example, instead of "Firstly, we adopt VMD technology...," the sentence could be revised to "Firstly, VMD technology is adopted...," maintaining a formal and objective tone more typical of academic writing.

Response: Thank the reviewers for their valuable suggestions. We have removed some colloquial expressions from the paper, making it more academic.

2.Answer：the comparison method used in previous studies is not well-established, as it is not discussed in the early related work analysis. It appears only in the evaluation section without proper definition or discussion. Providing a clearer explanation and contextualization of the comparison method would strengthen the methodological framework of the research.

Response: Thank the reviewers for their valuable suggestions. We have added descriptions of relevant methods in the previous work section and compared them, demonstrating that our combination algorithm has more advantages.

3.Answer： Further comments regarding comparison figures (e.g., Fig 9, 10, 12) were noted. The combination of multiple results in the same figure could make analysis challenging. Improvements such as using different colors or line styles/transparency may enhance clarity in visual representation.

Response: Thank the reviewers for their valuable suggestions. We have made modifications to the table, but the legend for the final result of the model run in this article is incorrect. We have now made the necessary changes, and we have included the comparison results of multiple models in one graph. However, some models have significant differences, which makes the graph not look very clear. I tried changing the color, but still in the current situation, so I have decided to continue using the original image. The final model run in this article is the main image, while other images are used for comparison.

4 Answer： Regarding the evaluation error index results, the presence of long decimal points raises questions about significance. Clarification on the significance of these decimal points in relation to the evaluation metrics would enhance the interpretation of the results.

Response: Thank the reviewers for their valuable suggestions. We have improved the final scoring result by retaining three decimal places, as the final r2 score had a different third decimal place. In order to make the comparison clearer, we have retained three decimal places.

|  | MAE | RMSE | R2 |
| --- | --- | --- | --- |
| LSTM | 378.162 | 517.581 | 0.530 |
| GRU | 376.910 | 511.652 | 0.562 |
| BERT^[22]^ | 377.852 | 511.728 | 0.547 |
| BP^[23]^ | 378.556 | 512.364 | 0.526 |
| CNN-LSTM^[24]^ | 358.586 | 485.351 | 0.585 |
| CNN-GRU | 356.253 | 477.598 | 0.599 |
| VMD-CNN-GRU | 55.285 | 75.308 | 0.993 |
| VMD-CNN-AM-GRU | 20.280 | 23.881 | 0.998 |

|  | MAE | RMSE | R2 |
| --- | --- | --- | --- |
| LSTM | 66.907 | 86.269 | 0.541 |
| GRU | 56.669 | 76.167 | 0.656 |
| BERT^[22]^ | 52.314 | 72.113 | 0.696 |
| BP[23] | 53.504 | 74.974 | 0.653 |
| CNN-LSTM[24] | 52.227 | 71.017 | 0.689 |
| CNN-GRU | 51.658 | 74.659 | 0.642 |
| VMD-CNN-GRU | 11.602 | 14.064 | 0.987 |
| VMD-CNN-AM-GRU | 6.095 | 8.340 | 0.995 |
